# Fair Play in a Context of Physical Education and Sports Behaviours

**DOI:** 10.3390/ijerph19042452

**Published:** 2022-02-21

**Authors:** Mateusz Ludwiczak, Małgorzata Bronikowska

**Affiliations:** Department of Recreation, Poznan University of Physical Education, 61-871 Poznan, Poland; bronikowska@awf.poznan.pl

**Keywords:** youths, Olympism, adolescents, prosocial competencies

## Abstract

The study examined prosocial behaviour and the perception of fair play in the context of physical education and sport (PES) in adolescents participating in sports, and those not undertaking sports at all. The aim of this study was to explore and indicate potential associations between an understanding of the concept of fair play and selected behaviours (e.g., abiding by the rules, obeying decisions of the referee and sportsmanship) in youth. In total, 1257 secondary school students, aged 16.1 ± 0.87 years (627 girls and 600 boys), were recruited. For assessing the level of fair play awareness (L), the Fair Play Moral Dimensions Concept Scale (FPMDCS) was used. To measure the declared level of prosocial behaviours of students, the survey My Physical Education Class (MPEC) was used. Participants were divided into groups in relation to training experience (no sports, amateur sports, professional sports) and subgroups, with levels according to their understanding of the Fair Play concept (L_1_ to L_3_). The results show significant difference in all dilemmas in various groups in relation to scores in subgroup L_3_ (*p* = 0.056; *p* = 0.012; *p* = 0.003) with regard to subgroups L_2_ and L_1_ in the level of understanding fair play. Overall, the percentage of respondents who view fair play only in the context of sport (L_1_) is 69%. Far fewer are those (31%) who understand it more broadly as a principle that applies to everyday life situations (L_3_). It seems to be crucial to promote moral development during PES activities, especially the value of respect.

## 1. Introduction

In recent years, it has increasingly been noted that school-aged students are less suited to situations that require social relationship competencies [1,2]. As a result, young people are spending less and less time with their peers, thus giving up the opportunity to build better social interactions [1]. It is therefore essential to create a favourable environment in PES contexts. It will allow youths to experience various situations and diverse dilemmas. However, it needs to be supported by prosocial reactions initiated by a PE teacher/sports coach, as well as being beneficial for promoting the ideals of sporting competition and moral behaviour. The Declaration on Sport, Tolerance and Fair Play agreed that sport is an important field of education [3]. Additionally, especially for young people, sport should serve primarily to convey universal values that help to raise the younger generation [2]. Sport, as a significant form of social activity, plays a key role on a global scale in the context of interpersonal communication and developing prosocial competencies [4,5,6,7]. As a universal phenomenon, sport affects all spheres of culture and, more importantly, educational systems and the values of young citizens and their behaviour [8]. Fair play as a common concept in sport ethics [9,10], one of the core principles of the Olympic Charter [11] and mission of the International Olympic Committee (I.O.C.), can also help in our understanding of justice in a broader social setting. Fair play should be considered not only in a sporting dimension. Additionally, as a part of everyday life, including all the desired behaviours of sportspersons in their social functioning [12]. On the other hand, unfortunately, sport in modern times is undergoing a crisis of values. With fouling now so fashionable, vicious and rule-breaking behaviour have, in recent decades, become not only visible and tolerated, but also something to be shown off [13]. Thus, it is important to prepare young people, skilfully and consciously, to serve their roles in adulthood fully and according to universal norms.

Age is one of the most critical variables to consider as moral judgment develops over time. A delay in the development of moral judgment leaves youths vulnerable, given increases in ego capabilities as well as peer stimuli from early through middle and late adolescence [14,15]. Moving from primary to secondary school leads the young individual into a new stage in the development of morality. Therefore, the individual begins to act according to the principles they recognize, regardless of the circumstances [16]. Satisfaction is gained by acting in accordance with standards, rather than by fear of punishment or the desire to receive a reward. Juveniles begin to consider the validity of actions taken and make their own moral judgments. Their moral judgments are based mostly on the intentions and motives of the offender.

A real dilemma, as a method of evaluating moral values based on hypothetical rather than theoretical dilemmas, can be of great environmental importance because participants can provide relevant situational content in relation to their everyday life and the cultural context that they experience [16]. To support the empirical validity of the theory, research has shown the hierarchical structure with objective measures of moral reasoning [17,18], which is crucial in accordance with cognitive development [19]. Reasoning is defined as a type of rationality directed towards deciding what to do and, when successful, issuing in an intention [20]. Hence, improvements in moral reasoning depend on instability in social interactions that result in a shift in perspective-taking.

Recent studies show that the influence of moral stages on the functioning of young people in life situations, such as mobbing at school, is negatively related to moral competencies [21]. There is evidence that adolescents are also characterized by a low level of moral competence, regardless of their participation in sports [22].

Moral principles are precursors to moral action, and the intention to perform an action, because they are motivational. More relevant models explaining moral behaviour include the four-component model of moral action [17], Bandura’s [23] social cognitive theory of moral thought and action, and the cognition model [24]. As Gibbons et al. [25] state, moral judgment and intentions are important indicators of one’s behaviour at a given moment. Moreover, Cheung et al. [18] showed that the higher the level of moral competence, the greater the positive influence on moral intentions. These findings confirm the view that moral awareness is motivational. Furthermore, awareness of personal interests is particularly responsible for immoral intentions. These results are consistent with the emphasis of cognitive-developmental theory on the cognitive basis of moral behaviour. The study clarifies the nature of moral behaviour as the embodiment of socially desirable principles—such as justice, social order and social progress—rather than as prosocial and altruistic behaviour centred around individual favour, e.g., being respectful to the teacher [26].

In a school setting, Olympic Education (OE) based on the neo-Olympism philosophy has been considered as a form of education [27,28,29,30]. An example of this might be also the Young Leaders Programme proposed by the IOC, which gives young people the opportunity to take responsibility for their environment. Through sport, they can develop the social dimension accordingly [31]. Whereas PES is a form of physical activity (PA) that allows children and adolescents to socialize and acquire social rules and values [32,33,34,35]. Studies on promoting Olympic values suggest an important and effective role of school in that process [27,28,29]. For instance, Parry [32] believes that the practice of sport, influenced by the philosophical anthropology of Olympism, offers a context and a route for PE teachers to achieve a number of important aims relating to moral education.

Ethics and education meet each other in the field of moral education. Most of the selected authors pointed out as valuable, albeit without carrying out further research and investigation on the matter [36,37,38]. Additionally, OE is a concept not only found within the Olympic Movement (IOC Agenda 2020 + 5 [39]. One recommendation to strengthen the role of sport is to promote Olympic values and social development programmes (including PE programmes for training teachers, sports coaches and volunteers). It is also becoming an integral part of the whole curriculum of elementary and secondary education in many countries [40,41,42]. The transfer of Olympic ethical values to education systems presents an opportunity to restore the position that PES deserves in general culture [13,28,43,44]. The need for OE results not only from the expectations of young people [32], but also from previous positive educational experiences in other countries like sport education [45,46]. For students who do not participate in sports, participation in PE classes is often the only way to learn fair play principals as a concept. Practicing sports, on the other hand, raises moral concerns for many of us.

Among the young, there is no difference in relation to gender in the understanding of the concept of fair play, in the context of participation in sport [42]. However, most young people perceive fair play only in the sphere of sport [47]. Values in sport may have important general implications for the intrinsic interest it provokes in participants. This analytic distinction covers most of the interpretations of the ideal and can serve as a normative starting point for further research.

Consequently, based on the abovementioned premises, we developed a study that aimed at exploring and indicating potential associations between levels of understanding of the concept of fair play and selected behavioural dilemmas (i.e., abiding by the rules, obeying decisions of the referee and sportsmanship) in young adolescents in the PES context. For that reason, we proposed two hypotheses: firstly, the teaching process of PES causes an increase in the level of positive prosocial behaviours and allows for a better understanding of the phenomenon of fair play among adolescences; secondly, despite different involvement in sports secondary school, students with the highest level of fair play indicate more positive prosocial behaviours in the context of the selected dilemmas.

## 2. Materials and Methods

### 2.1. Participants and Procedure

This study was conducted in the 2018/19 school year. It included 1257 secondary school students (no sports *n* = 197, amateur sports *n* = 496, professional sports *n* = 562) aged 16.1 ± 0.87 years (657 girls; 600 boys), recruited from 9 randomly selected schools in three major cities in the Wielkopolska Province in Poland. For the purpose of the study, we have proposed the following definitions: professional participation in sport meant engagement in regular system of competitions, organized by a sports federation [48], while in case of amateur sport, the most widespread definition is based on those individuals who practice sport casually as a hobby, for pure pleasure [49]. Permission for carrying out the research was secured separately and granted by the institutions concerned. The sample unit was a randomly selected school class. All participants taking part in an ‘in-person’ examination were informed about the study’s aims, risks and benefits, and parental consent was obtained before the study began. Questionnaires were completed in whole-class groups during a regular school lesson in quiet classroom conditions and took approximately 30 min to complete. All respondents were notified that their involvement would be anonymous and voluntary, and the fact that the results would only be used for scientific purposes. The investigator followed the study to assess the exposure and outcomes over a longer period of time [50]. This approach was aimed at defining the current relationships between the examined concepts in this study.

A cross-sectional study design was used. The research study was performed using the diagnostic survey method with the use of a structured survey questionnaire previously prepared, tested and assessed by competent evaluators. Those who agreed to participate in the project were requested to read and complete the inquiry form during regular school classes. Moreover, the respondents were informed of the option of withdrawing in case where they felt the need not to answer certain questions included in the questionnaire. The total return rate was 94%.

### 2.2. Fair Play Moral Dimensions Concept Scale (FPMDCS)

For assessing the level of fair play awareness (L), we used the three-level scale known as the Fair Play Moral Dimensions Concept Scale [42], designed based on Parry’s Fair Play Moral Dimensions Theory [11]. The scale contains three statements about the understanding of the concept of fair play, related to the levels (L_1_, L_2_, L_3_). The first level of fair play awareness (L_1_) is limited to the application of previously set/codified sporting rules during a sports competition (i.e., compliance with the rules that are the basic guideline for any sport and any kind of competition) [42]. The second level of fair play awareness (L_2_) refers not only to the application of codified sporting rules during a competition, but also to the employment of a code of behaviour resulting from the professional etiquette of those participating in competitive sport (i.e., efforts to compete in the right spirit, which means obeying written law that corresponds to generally accepted moral values) [42]. The third level in the application of the principle of fair play (L_3_) defines all the desired behaviours of the sportspersons by which they will stand out, not only in their sporting life, but also in their everyday life (i.e., fair play manifests itself in all situations as a life attitude, it is firmly anchored in the hierarchy of universal values) [42]. Responders were requested to mark one of the levels in accordance with their understanding of the principle of fair play, based on their own knowledge and previous experience. A conducted test–retest (following a two-week break) indicated a level of reliability of 0.78.

### 2.3. My Physical Education Class (MPEC)

To measure the declared prosocial behaviour of secondary school students, the My Physical Education Class (MPEC) questionnaire [27] was used, which demonstrated internal consistency (0.75) in an earlier study of a similar population [22]. The questionnaire is based on Horrocks’ [51] Pro-social Play Behaviour Inventory (HPPBI) and includes 10 moral dilemmas concerning sportsmanship behaviours, i.e., teasing others, showing off, arguing with teammates, complaining, sharing equipment, disobeying the rules of the game, ‘hogging’ the ball, disputing officials’ decisions, not taking turns and ignoring teammates’ suggestions for improving—all situated in the context of PES.

For this study, three dilemmas that are most closely related to the concept of fair play (teasing others, disobeying the rules of the game and disputing officials’ decisions) were selected, e.g., “When you win while playing in PE classes, you wonder whether to show off to your friends”. The questionnaire measures moral judgement, reasoning and the intentions of an individual through a self-rating system. Judgment and intentions are scored on a three-point Likert scale, and reasons on a five-point Likert scale, ranging from 1 = not at all to 5 = very much.

### 2.4. Ethics

The study was approved by the Bioethics Committee of the Karol Marcinkowski Medical University, Poznan (No. 893/18).

### 2.5. Statistic

Due to the lack of a normal distribution in the analysed groups, non-parametric statistical tools were employed. The chi-squared test was used to examine the independence of the qualitative factors from the type of group being examined, namely, those not practicing sport and those practicing sport at an amateur or professional level. The percentage distribution of indicators in the groups presented in tables also contains chi-squared statistical values and probability. If *p* < 0.05, the distribution of a given factor was dependent on the group, with it then becoming interesting in which categories a clear difference in the observed percentage would appear. A two-proportion z-test was used to test for a difference between two population proportions (differences between subgroups, i.e., L_1_–L_2_, L_1_–L_3_, L_2_–L_3_). All statistical analyses were completed using the STATISTICA ver. 13.1 software package (Stat Soft, Krakow, Poland).

## 3. Results

Table 1 presents the differences between levels of comprehension of the concept of fair play among students in relation to the dilemma, “When you win while playing in PE classes, you wonder whether to show off to your friends”, and at the same time the difference between subgroups, in terms of moral judgement, reasoning and intentions regarding their involvement in sports training (no sports training; amateur training; professional training).

Concerning the first question, in the ‘judgment’ category, data analysis indicates a tendency to significant difference in the group of professional sports-player responders (*p* = 0.056).

Participants from the L_3_ subgroup returned a higher result for the answer “it is not okay to show off in PE class” (58%), indicating the appropriate judgment in this specific moral context, in comparison to subgroups L_2_ (44%) (*p* = 0.011) and L_1_ (47%) (*p* = 0.056). Among the other groups—no sports and amateur sports—there were no significant differences among subgroups.

Analysis of the second question, relating to ‘reasoning’ as a component of the first dilemma, did not show any statistically significant differences in any group. The most frequent declaration among all surveyed groups at all levels of understanding of the concept of fair play was the response: “it is nice or not nice to show off”.

The analysis of the study results for the third question, in the ‘intentions’ category of the first dilemma, did not indicate a statistical difference in the declarations of any of the study groups. Levels of fair play did not affect the differences between participants in relation to prosocial behaviours. All groups of students mostly declare that they will “never show off” in future PE class, which is believed to be the model attitude. In this dilemma, the vast majority of professional athletes responded to the moral judgement aspect with appropriate sportsmanship behaviours.

Table 2 presents the difference between the levels of comprehension of the concept of fair play among students in relation to the second dilemma, which concerns the declared attitude of the respondents in the context of following the rules of the game when playing in PE class. Likewise, in Table 1, the differences between subgroups were also presented to see if there are any significant differences.

Analysis of the research results concerning the first question, in the ‘judgment’ category, indicates statistical significance among amateur sports-players (*p* = 0.012). Most participants in all subgroups declared “it is not okay not to follow the games rules during PE lessons” as an answer to this dilemma, though students from subgroup L_1_ (60%) and L_2_ (61%) returned a statistically significant lower percentage of such responses than did subgroup L_3_ (74%) (L_3_–L_1_; *p* = 0.011; L_3_–L_2_; *p* = 0.011). Subgroup L_3_ also returned a lower percentage (8%) of the reverse answer “it is okay not to follow the games rules during PE lessons” among amateur sports-players in comparison with subgroups L_2_ (11%) and L_1_ (18%) (L_3_–L_1_; *p* = 0.001; L_3_–L_2_; *p* = 0.001).

In the ‘reasoning’ category of the second dilemma, there were no statistically significant differences in any of the levels of understanding of the principle of fair play. In all groups, participants tended to the response “it is fair or not fair to follow the rules”.

Analysis of the results for the third question, in the ‘intentions’ category of this second dilemma, indicates marginal significance in the no-sports group (*p* = 0.059). L_3_ participants declared that in future, they will “never disobey rules” (61%). This was a significantly higher result than among students from L_2_ (53%) and L_1_ (60%). In the L_3_ subgroup, only 3% declared that they will “disobey rules most of the time”. Groups of amateur and professional sports-players also declared prosocial attitudes in relation to disobeying rules. In relation to dilemma, amateur athletes maintained similar reactions across all levels of understanding of fair play. In intentions of students not practicing sport could noticed a positive outcome in relation to moral behaviours.

Table 3 presents the difference between levels of comprehension of fair play among students in relation to the dilemma concerning the student’s declarative attitude towards a decision made by the referee while playing in PE (i.e., to question the decision or not) as well the difference between subgroups to show significant differences.

Analysis of the research results concerning the first question, in the ‘judgment’ category, indicates a statistical difference in the group of professional sports-players (*p* = 0.003). In this group, students from the subgroup with the strongest understanding of the principle of fair play (L_3_) declared that it is “not OK to dispute a referee’s decision in PE class” at a higher percentage (28%)—statistically significant in relation to subgroup L_1_ (17%) and L_2_ (20%). In subgroup L_1_, the percentage answering that “it is OK to dispute a referee’s decision in PE class” (27%) was even higher than the percentage answering that “it is not OK to dispute a referee’s decision in PE class” (17%).

In the analysis of the second question of the third dilemma, concerning ‘reasoning’, a statistical difference was noticed among amateur sports-players (*p* = 0.003). In subgroup L_1_, the most common answer was “get punished” (43%); in L_2_ it was “against the rules” (31%); students from L_3_ returned a higher result (32%) for the answer “is it fair or not to dispute a referee’s decision in PE class”, indicating hesitation that might be caused by specific environmental conditions (e.g., upbringing, experience, knowledge, etc.).

As shown by the above analysis, results concerning the ‘intentions’ category for the third dilemma did not indicate a statistical difference between any of the levels of understanding of fair play principles. There is a clear tendency, however, to intend to make decisions about disputing with the referee “sometimes”, leaving the decision to circumstances (48–62% through all stages of fair play understanding in all study-groups). On the other hand, the low percentage (24–37%) of those declaring compliance with the referee’s decision during the game in any situation seems disturbing. Assessing the dilemma concerning the acceptance of referee decisions, professional sports-players indicated an attitude undermining the role of a referee.

## 4. Discussion

Sport has influence on social skills and, used properly, could teach competition, cooperation, role-playing and discipline regarding rules, regulations and goals [52]. It can be also seen as a laboratory of human experience [38]. Sport is supposed to create a system of value based on the experiences of participants via training. PE as a school subject consists of the core principles of sport and provides an opportunity to develop students, not only physically, but also morally and socially. Therefore, a part of the didactic process in many countries is OE, with particular emphasis on the role of fair play as the overriding and universal principle [13,30,32,41,43,44]. It is assumed that fair play as a part of OE should develop moral competencies in terms of judgment, reasoning and intentions [44]. According to our hypothesis the results of this study show that the level of understanding fair play has a crucial role in behaviour related to moral dilemmas. In each situation participants from subgroup L_3_, with the highest understanding of the concept of fair play, showed a higher level of positive prosocial behaviour in relation to judgment, reasoning and intensions in comparison to students from subgroups L_1_ and L_2_ (representing the lowest and moderate levels of understanding of fair play). The fair play attitude and awareness influences prosocial behaviour and relations in teams on and off the field [53], and also reduces antisocial behaviour among adolescents [54]. Similar results were presented by Vidoni and Ward [55], who claim that socially competent players support each other’s performance, show respect to opponents and officials and, furthermore, accept losses without complaint and win without gloating. On the contrary, antisocial behaviours (e.g., cheating, gamesmanship, doping) violating the rules or the spirit of the game have become a part of the game [56], with those athletes using various forms of moral disengagement to differentiate between deception and ‘clever’ play, or which can lead to other unwanted antisocial behaviours in school, such as bullying or cheating [42]. In our study, in the first and second dilemmas, we noticed well-represented prosocial behaviours among most participants in all variables. By contrast, in the third dilemma relating to referees’ decisions, we observed that a majority of participants were uncertain in their declaration, contrasting with Vidoni and Ward’s study [55] in which athletes showed respect to officials. We must, therefore, note that sports participants might be influenced in their moral development in both a positive and a negative way [57]. For this reason, PE teachers and sport coaches, through their teaching/coaching style, play a crucial role in the upbringing and sound education of the younger generation [58]. Young people who are socially competent react properly to situations occurring in everyday life by managing conflicts and dealing appropriately with any disagreements [59]. With this in mind, it seems important to know how students perceive fair play and if it influences their experiences, in a way related to PE class. In the case of PE, the problem may be rooted in a still-present misconception in understanding the modern paradigm of PE, namely, in that a traditional physical sport-centred approach is still more present than a holistic approach, which would focus on enhancing social attitudes [60], moral development, and life-long learning skills [22]. Kowalska [61] noticed that changes in behaviour in relation to sports situations also change behaviour in everyday life dilemmas. At the same time, she claims that, for a minor group of students (26.2%), fair play is more than just a sports principle [47]. In her study, half of the responders believed that fair play is respected by people, regardless of whether they participate in sports or not. Conversely, 31% of young respondents did not see any relation between fair play and everyday life situations. Comparing the above-mentioned conclusions to our research, we can highlight the number of participants who perceive fair play either only in the context of sport settings (69%) or more broadly as a rule concerning situations in everyday life (31%).

Among young people in sport, the most frequent display of sportsmanship [62] is the players’ greeting after the game. This confirms the right attitude towards other players and recognizes showing off as an undesirable behaviour. Peer violence has a major impact on the self-esteem of young people [63], and practicing sports in an appropriate manner could reduce this problem [63]. Some studies indicate that young athletes, in comparison to non-sporting school students, have lower levels of aggression [63], therefore behaving properly both within and beyond sporting situations. This outcome corresponds with our research. The results show that students participating in professional sports training understand fair play, not only in sport, but also beyond sports situations. They are in line with other authors [42,47].

Previous research [64,65] has shown that violence against referees has become more common, which can lead to dismissive behaviour in relation to umpires. Our results show that most young people consider undermining a referee’s decision even during PE classes. Krebs and Denton [66] argue that people invoke moral judgment afterwards to justify what they have done. Furthermore, these authors claim that moral judgment stages are only weakly associated with behaviour and may be used in a flexible way in accordance with different social contexts or reasons. They argue [66] that optimal moral judgment does not reflect adequacy but usefulness, that is, ‘creating the conditions that enable people to achieve their goals and advance their interests in cooperative ways’ [66] (p. 646). In fact, different kinds of contexts pull toward different forms of moral judgment. In this sense, people move in and out of moral orders, not stages of moral development. Other authors [67,68] show interesting examples of flexible stage use, providing evidence that higher moral judgment competence increases resistance to low-stage pulling contexts or sets the stage for moral action. In our study, the difference among students is related to reasoning about dilemmas and depends on the level of understanding of the concept of fair play. In terms of moral reasoning related to the decisions of referees, the results raise our curiosity. Among amateur sports-players, each fair play comprehension level showed a different reasoning pattern. This might be associated with the developing brains of adolescents, information processing capacity and working memory can be disturbed by a growing reward and control system, leading to risk-taking or unethical behaviours [69]. These limitations have not always been challenged effectively, particularly in terms of PES. Shields and Bredemeier [28], in their research on school sport, claim that PES, if aimed at ‘winning at all cost’, may cause problems such as cheating, arguing with officials and egotistical celebration. In relation to ‘game reasoning’ [28], players react with a lower moral reasoning stage during participation in sport, but, when they understand that issues are related to everyday life, they shift to more mature reasoning. In our results for the dilemma relating to referee decisions, in the reasoning aspect, we can see some correlation between answers at each level of fair play comprehension and the stages of Kohlberg’s scale [13]. For students from subgroup L_1_ the most frequent answer was “get punished”, whereas, for subgroup L_2_, it was “against the rules” and, amongst participants representing the highest comprehension group, subgroup L_3_, “fair or not fair” was the most declared statement. These results align with previous research by Shields and Bredemeier [28] and Bronikowska et al. [22].

The explanation of our results might be that the process of learning values in that group of school students was not conducted appropriately and most probably lacked guidance by established theories, such as those of Koh [70] or Kohlberg [14], or may not have been trained at all. In addition, differences between sports education in PE contexts and in extra sports activities, written about successively by Vella et al. [71] and Lee [72], may have affected our results. Situated within this context, our results may come as no surprise, but they do call for more training on moral issues for students during school years, especially since previous research [64,65] showed that this is not a problem only in youth sport but across all age categories in sporting competitions. This aspect is worth deeper consideration in future.

Some limitations of this study need to be acknowledged. There are cognition measures (multiple-choice questionnaires to evaluate moral judgment representing different stages) using hypothetical dilemmas (moral competence, not moral performance). These factors might affect the findings. The study was based on a declarative assessment of the participants’ attitudes, whereas real-life situations might be a valuable check for participants’ real values. Hence, our next step in further research will be to design an adequate intervention programme, considering the dilemmas and factors affecting evaluation in the PES environment.

## 5. Conclusions

This study showed that students recognized dilemmas mostly with prosocial behaviour and that this was dependent on the presented level of fair play amongst responders in relation to moral judgement, reasoning and intentions. In the dilemma concerning relations with classmates, the vast majority of professional athletes responded to the moral judgement aspect with appropriate sportsmanship behaviours. Moreover, in the dilemma concerning abiding by the rules, amateur athletes maintained similar reactions across all levels of understanding of fair play. In the same dilemma, the intentions of students not practicing sport showed a positive outcome in relation to moral behaviours.

In the last dilemma, concerning the acceptance of referee decisions, professional sports-players indicated an attitude undermining the role of a referee. Previous research [65,66] showed that this is not a problem only in youth sport but across all age categories in sporting competitions. This aspect is worth deeper consideration in future. In terms of moral reasoning related to the decisions of referees, the results raise our wonder. Among those who declared themselves as amateur sports-players, each fair play comprehension level showed a different reasoning pattern.

It seems to be crucial to promote moral development during PES activities, especially respect for other people—teammates, and the referee. It is worth emphasizing that an understanding of fair play determined/mitigated outcomes in all dilemmas in each group of students. Therefore, in the process of education, fair play should be properly related to the principles of functioning in everyday life. Young people need a sense of understanding and a suitable level of awareness in order to get involved in undertaking a life-long PA.

It is also worth considering the creation of a pedagogical intervention which could help students to develop their moral competencies and prosocial behaviours, in relation to recent research and the low level of moral competence among the young. As a final remark, we should say that educating one to practice a behaviour of fair play as one of the most universal principles in sport and that goes beyond sport is, or at least should be, one of the most fundamental duties of modern education, not just through school PE classes, but throughout the schooling system. This calls also for adjustments in curricula aimed at suitably preparing future teachers for the proper implementation of this ideal in the school environment, where the sensitization of young people to the concept of a ‘clean game’ during school studies will hopefully shape their moral outlooks and have an impact on their adult lives in a fundamental manner.

## Figures and Tables

**Table 1:** Student attitudes to peer relations during PE classes, considering their level of involvement in sporting activities and their level of comprehension of the concept of fair play (%).

Dilemma 1	When You Win while Playing in PE Classes, You Wonder whether to Show Off to Your Friends.								
	**No Sports *n* = 199**			**Amateur Sports *n* = 496**			**Professional Sports *n* = 562**		
Level of FP (L)	L_1_ *n* = 52	L_2_ *n* = 85	L_3_ *n* = 62	L_1_ *n* = 127	L_2_ *n* = 205	L_3_ *n* = 164	L_1_ *n* = 140	L_2_ *n* = 262	L_3_ *n* = 160
Judgment	Do you think that it is OK to show off in PE class?								
Ok	10	8	13	17	13	9	12	14	13
Sometimes	38	48	31	31	34	39	41	42	29 *,^B,C^
Not Ok	52	44	56	52	53	52	47	44	58 *,^B,C^
Ch^2^ (*p* value)	4.87 (0.300)			5.27 (0.259)			9.17 (0.056)		
Reasoning	Which is the most important thing to consider when you decide whether it is OK to show off?								
Get punished	8	5	5	10	5	4	7	5	7
Get even	9	6	8	13	10	10	10	16	10
Nice or not nice	44	48	42	43	52	44	53	42	49
Against rules	8	7	5	6	6	5	6	6	4
Fair or not	31	34	40	28	27	37	24	31	30
Chi^2^ (*p* value)	2.73 (0.948)			11.71 (0.164)			9.5 (0.300)		
Intentions	If you win games in future PE class, what do you think you will do?								
Never show off	65	58	68	66	61	63	57	54	62
Sometimes	29	37	24	24	33	31	36	38	28
Show off most of the times	6	5	8	10	6	6	7	8	10
Chi^2^ (*p* value)	3.53 (0.472)			4.42 (0.350)			5.62 (0.223)		

* Notes significant difference between subgroups, *p* < 0.05, L_1_–L_3_ ^B^, L_2_–L_3_ ^C^.

**Table 2:** Student attitudes during PE classes in relation to obeying the rules of the game, with respect to their level of involvement in sporting activities and their level of comprehension of the concept of fair play (%).

Dilemma 2	When You Participate in Different Games during Your PE Classes, You Wonder whether to Follow Their Rules.								
	**No Sports *n* = 199**			**Amateur Sports *n* = 496**			**Professional Sports *n* = 562**		
Level of FP (L)	L_1_ *n* = 52	L_2_ *n* = 85	L_3_ *n* = 62	L_1_ *n* = 127	L_2_ *n* = 205	L_3_ *n* = 164	L_1_ *n* = 140	L_2_ *n* = 262	L_3_ *n* = 160
Judgement	Do you think it is okay not to follow the games rules during PE lessons?								
Ok	21	17	8	18	11	8 *,^B,C^	15	15	15
Sometimes	25	22	15	22	28	18 *,^C^	26	24	26
Not Ok	54	61	77	60	61	74 *,^B,C^	60	61	59
Chi^2^ (*p* value)	7.80 (0.098)			12.77 (0.012) *			0.27 (0.991)		
Reasoning	Which is the most important thing to consider when you decide whether it is OK to follow the rules?								
Get punished	25	19	18	20	14	11	34	18	15
Get even	4	1	7	6	4	4	11	7	8
Nice or not nice	14	17	15	17	16	20	19	13	15
Against rules	21	28	19	27	23	21	21	18	22
Fair or not	37	35	42	30	42	44	29	44	40
Chi^2^ (*p* value)	5.37 (0.716)			11.33 (0.181)			12.56 (0.127)		
Intentions	If you are playing games in future PE classes, what do you think you will do?								
Never disobey rules	60	53	61	54	58	68	50	60	57
Sometimes	35	38	36	37	35	25	42	34	38
Disobey rules most of the time	6	9	3 *,^B,C^	9	7	7	8	6	5
Chi^2^ (*p* value)	2.75 (0.059)			7.20 (0.125)			4.49 (0.343)		

* Notes significant difference between subgroups, *p* < 0.05, L_1_–L_3_ ^B^, L_2_–L_3_ ^C^.

**Table 3:** Student attitudes to the referee’s decision made during the PE lesson, considering their level of involvement in sporting activities and their level of comprehension of the concept of fair play (%).

Dilemma 3	When a Referee Decides during a Game in Your PE Class, You Wonder whether to Dispute the Decision.								
	**No Sports *n* = 199**			**Amateur Sports *n* = 496**			**Professional Sports *n* = 562**		
Level of FP (L)	L_1_ *n* = 52	L_2_ *n* = 85	L_3_ *n* = 62	L_1_ *n* = 127	L_2_ *n* = 205	L_3_ *n* = 164	L_1_ *n* = 140	L_2_ *n* = 262	L_3_ *n* = 160
Judgement	Do you think that it is OK to dispute a referee’s decision in PE class?								
Ok	19	17	15	24	19	18	27	16	23
Sometimes	69	66	60	54	60	56	56	65	49
Not Ok	12	18	26	22	22	26	17	20	28 *,^B,C^
Chi^2^ (*p* value)	3.97 (0.409)			2.81 (0.589)			15.68 (0.003) *		
Reasoning	Which is the most important thing to consider when you decide whether to dispute a referee’s decision?								
Get punished	37	24	27	43	28 *,^A^	24 *,^B^	41	31	33
Get even	10	8	0	2	7	5	8	6	4
Nice or not nice	14	15	21	16	11	15	14	13	16
Against rules	23	26	21	19	31 *,^A^	24	29	29	23
Fair or not	17	27	31	20	24	32 *,^B^	21	21	23
Chi^2^ (*p* value)	9.23 (*p* = 0.322)			22.11 (0.003) *			11.76 (0.162)		
Intentions	If a referee makes decision in future PE classes, what do you think you will do?								
Never dispute	23	17	24	23	14	19	20	23	17
Sometimes	50	48	48	53	62	55	49	57	50
Dispute most of the times	27	35	27	24	24	26	31	37	33
Chi^2^ (*p* value)	2.31 (0.678)			5.67 (0.224)			0.60 (0.962)		

* Notes significant difference between subgroups, *p* < 0.05, L_1_–L_2_ ^A^, L_1_–L_3_ ^B^, L_2_–L_3_ ^C^.

## Data Availability

The data that support the findings of this study are available from the corresponding author, upon reasonable request.
